# Efficacy and Safety of Human Chorionic Gonadotropin Monotherapy for Men With Hypogonadal Symptoms and Normal Testosterone

**DOI:** 10.7759/cureus.25543

**Published:** 2022-05-31

**Authors:** Isaac Zucker, Quinn Rainer, Raghav K Pai, Ranjith Ramasamy, Thomas A Masterson

**Affiliations:** 1 Medicine, Florida International University, Miami, USA; 2 Urology, University of Miami, Miami, USA

**Keywords:** eugonadism, testosterone deficiency, hypogonadism, testosterone therapy, human chorionic gonadotropin

## Abstract

Background

Male hypogonadism has a prevalence of about 6% and is defined by two-morning testosterone levels below 300 ng/dl associated with symptoms. This definition presents a challenging problem for patients without other medical problems but with symptoms of low testosterone (T) who do not meet the biochemical criteria for therapy.

Objectives

Our objective was to evaluate changes in symptoms and side effects in men with T levels >300ng/dL using human chorionic gonadotropin (hCG) monotherapy for the treatment of hypogonadal symptoms.

Methods

After IRB approval, 31 male patients treated with hCG monotherapy for low T symptoms were retrospectively reviewed. We evaluated changes in hormones, hypogonadal symptoms, and the incidence of thromboembolic events before and after starting hCG.

Results

We found subjective improvement in erectile dysfunction, 86% (19/22), and libido, 80% (20/25), with no patient experiencing a thromboembolic event. In addition, no change was observed in the follicle-stimulating hormone, luteinizing hormone, estradiol, hematocrit, hemoglobin A1c, and prostate-specific antigen.

Conclusion

Weekly treatment with hCG appears safe and can improve hypogonadal symptoms in patients with T >300 ng/dl without changes to hematocrit, prostate-specific antigen, and hemoglobin A1c.

## Introduction

Testosterone deficiency (TD) is a clinical syndrome caused by the gonads' inability to produce adequate levels of circulating testosterone [1,2]. TD is defined as having morning serum testosterone (T) levels consistently below 300ng/dl, with associated symptoms such as diminished libido, erectile dysfunction, weight gain, fatigue, poor concentration, and depression [3]. Testosterone deficiency has a prevalence of approximately 6% and increases with age [4]. Testosterone replacement therapy (TRT) with exogenous testosterone is the mainstay of treatment for men with TD and is indicated only for men meeting both biochemical and symptomatic criteria. Men with testosterone levels above 300 with symptoms of low testosterone represent a challenging patient population, as there is no clear guidance on treatment, and physicians often encounter patients with testosterone levels that are "low for their age".

Human chorionic gonadotropin (hCG) is homologous to luteinizing hormone (LH) and stimulates endogenous testosterone production from the testes. The American Urological Association (AUA) recommends using hCG for men with TD and fertility concerns, as it can maintain sperm production, while exogenous testosterone can act as a contraceptive. There is limited research on the efficacy and safety of hCG monotherapy, particularly in men who fail to meet the biochemical criteria for conventional TRT [5]. As a result, we sought to evaluate the response of patients to hCG monotherapy. We hypothesize that hCG is a safe and effective therapy for treating hypogonadal symptoms in men with T levels >300ng/dL who do not meet the criteria for TRT.

Objective

Our objective was to evaluate changes in symptoms and side effects in men presenting with hypogonadal symptoms and baseline T levels >300ng/dL using hCG monotherapy.

## Materials and methods

The study was conducted after the institutional review board from the University of Miami (IRB) approved for retrospective chart review. We retrospectively assessed the charts of 31 men (age 25-79) who began hCG monotherapy between October 2017 and August 2020, treated by one andrologist at the University of Miami for hypogonadal symptoms with a T average >300 ng/dL, who had laboratory investigations and clinical appointments at least one month after the initiation of hCG therapy. Patients were treated with varying doses of hCG as determined by an andrologist (range: 1000-3000 international units (IU) twice a week). All patients had two initial T tests, and the median was used to establish a baseline. All men were screened for other causes of symptoms, including pituitary adenomas, obstructive sleep apnea, depression, and thyroid disorders. We evaluated changes in hormones: T, LH, follicle-stimulating hormone (FSH), estradiol (E), hematocrit (HCT), glycated hemoglobin (A1c), and prostate-specific antigen (PSA).

On the initial visit, all patients were screened for symptoms of hypogonadism, including low libido and erectile dysfunction (ED). At each follow-up appointment, we asked patients to report subjective changes in hypogonadal symptoms to assess for symptom improvement. Follow-up times were variable depending on doctor recommendation and hCG dosage. As there are no recommended validated questionaries to screen or monitor symptom improvement [1], subjective improvement of specific hypogonadal symptoms as identified when initially prescribed hCG was recorded and documented. In subsequent follow-up visits, all patients were asked for specific improvements in symptomatology previously expressed. Finally, the patient's treatment start date was recorded, and the latest follow-up visits were made to evaluate the duration of treatment and patient side effects, including stroke, deep vein thrombosis, myocardial infarction, and change in hCG dose.

Statistical analysis

All results are presented as medians with an interquartile range. The Mann-Whitney U test was used to compare pre- and post-treatment values, and significance was set at p=0.05. All patient personal health information was completely de-identified for analysis. Only patients with completed pre and post-lab values were included in the analysis for changes in hormones. All statistical analysis was completed in Excel (Microsoft, Redmond, Washington).

## Results

The median age of patients was 52 (21.5) years old, with a BMI of 28.1 (6.6) kg/m^2^ (Table 1). The average follow-up after starting hCG therapy was 41.7 weeks, ranging from 11 to 122 weeks. The average hCG dosage was 1529.03 IU. There was no significant change in serum T (413 (143.1) ng/dL to 433 (174) ng/dL), FSH (3.1 (2.65) to 3.05(2.0) mIU/mL), PSA (1.35 (1.18) to 1.53 (2.0) ng/mL), HCT (42.85 (2.85) to 44.85 (2.7) %), Estradiol (27.5 (4.35) to 32 (8.9) pg/mL), A1c (5.85 (0.9) to 5.95 (0.6) %) or LH (4.8 (3.4) to 4.0 (2.2) mIU/mL) (Table 2). When evaluated for improvement of ED and low libido, 86% (19/22), and 80% (20/25) of patients reported improvement of each symptom, respectively (Table 3). All patients with ED were noted to be on another medication or therapy specifically for ED. No thromboembolic events or hCG side effects, including headache, gynecomastia, and gastrointestinal issues, were observed (Table 3).

**Table 1:** Patient demographics (n=31)

Patient demographics	n (%)
Median age in years (IQR)	52 (21.5)
Body mass index in kg/m^2^	
<18.4	0 (0)
18.5-24.9	8 (25.8)
25-29.9	11 (35.5)
>30	10 (32.3)
Unknown	2 (6.5)
Race	
White	30 (96.8)
African American	1 (3.2)
Ethnicity	
Hispanic	17 (54.8)
Non-Hispanic	14 (45.2)
Patient symptoms	
Erectile dysfunction	22 (70.9)
Low libido	25 (80.6)

**Table 2:** Median values before and after hCG therapy hCG - human chorionic gonadotropin

	Before hCG treatment		After hCG treatment			
	Median	Interquartile range	Median	Interquartile range	p-value	
Testosterone (ng/dl)	413.0	351.9-495.0	433.0	359.0-533.0	0.57	
Follicle-stimulating hormone (mIU/mL)	3.1	2.1-4.8	3.1	2.1-4.1	0.91	
Luteinizing hormone (mIU/mL)	4.8	3.6-6.9	4.0	2.4-4.6	0.14	
Estradiol (pg/mL)	27.5	26.9-31.2	32.0	34.5-8.8	0.32	
Prostate specific antigen (ng/mL)	1.4	0.7-1.9	1.5	0.8-2.7	0.92	
Hematocrit	42.9	42.3-45.2	44.9	43.8-46.4	0.19	
Hemoglobin A1C	5.9	5.6-6.5	6.0	05.8-6.4	0.69	

**Table 3:** Patient response and side effects to hCG therapy hCG - human chorionic gonadotropin

Side effect	n (%)
Erectile dysfunction	19/22 (86%)
Low libido	20/25 (80%)
hCG side effects	
Gynecomastia	0/31 (0%)
Injection site pain	0/31 (0%)
Nausea/vomiting	0/31 (0%)
Headache	0/31 (0%)
Thrombotic events	0/31 (0%)

## Discussion

For patients with T levels <300ng/dL and associated symptoms, the AUA recommends TRT; however, in patients who do not meet biochemical criteria, limited options exist [1]. As a result, we retrospectively analyzed 31 charts of men treated with hCG monotherapy for hypogonadal symptoms and evaluated changes in hormones, symptoms, and side effects. We found that hCG monotherapy improved hypogonadal symptoms without the undesirable side effects of hCG (gynecomastia, headache, and gastrointestinal complaints) or exogenous testosterone (polycythemia or thromboembolic events) [6,7]. While we did not observe a significant increase in testosterone level, previous literature shows that hCG was able to increase patient T levels; therefore, the lack of increase in T levels could be attributed to the low dose of hCG in our study [8-10]. 

The use of hCG has historically been recommended for men with TD interested in maintaining fertility or recovering sperm production in men previously on exogenous testosterone [5, 10, 11]. Several studies support hCG therapy to recover spermatogenesis after testosterone use but do not report the effect of hCG on symptoms or side effects [11]. In addition, hCG has been utilized in patients with isolated hypogonadotropic hypogonadism with a significant response to testicular volume, testosterone levels, and sperm production [9]. Finally, there has been conflicting literature that has shown that clomid or anastrozole may or may not benefit patients with hypogonadal symptoms in men with T<300 ng/dL [12-14]. All of the studies above explore the use of hCG for fertility and sperm production; however, its use in hypogonadism has not been elucidated. 

Few studies have reported hCG effect on symptoms of hypogonadism. Buvat et al. showed that in a limited subset of patients with psychogenic ED and lack of sexual desire that one month after treatment with hCG, there was improvement in symptoms, and the change in T did not correlate with an increase in T [15]. Building on this, Ishikawa found that in hypogonadal men (T<300), hCG was effective at improving erections [16]. Finally, in patients who were previously on TRT, hCG monotherapy was shown to improve erections and libido without change in T levels [17]. Previous literature has also shown a strong association in patients <40 years with T <400 ng/dL may have hypogonadal symptoms; however, they would not qualify for treatment under the AUA guidelines [18]. Therefore, our study is the first to identify hCG as a possible therapy for patients with hypogonadal symptoms and normal T.

Limitations of this study include the retrospective design, small sample size, short follow-up period after hCG administration, and the varying dosages of hCG. In addition, there is a lack of empirical measures to assess changes in energy level, and as a result, patients' energy could not be included in the final analysis. Strengths of the paper include that, to the best of our knowledge, this is the largest cohort studying the effect of hCG on hypogonadal symptoms in men with testosterone levels >300ng/dL. Additionally, all patients had complete data to assess before and after starting hCG. Despite these limitations, our study adds to the literature suggesting that hCG monotherapy is safe and effective in managing hypogonadal symptoms. 

## Conclusions

Our results indicate that hCG monotherapy appears to safely improve hypogonadal symptoms, even with baseline T levels >300 ng/dL. Although we did not observe a statistically significant change in T levels, patients reported symptomatic improvement without significant side effects or changes in HCT, PSA, A1c, FSH, or LH, and no thromboembolic events were recorded. Further study is needed in a large randomized blinded fashion, with validated questionnaires, to determine the true efficacy of hCG monotherapy in the management of patients with hypogonadal symptoms and normal T levels.

## References

[1] Mulhall JP, Trost LW, Brannigan RE (2018). Evaluation and management of testosterone deficiency: AUA guideline. J Urol.

[2] Bhasin S, Cunningham GR, Hayes FJ, Matsumoto AM, Snyder PJ, Swerdloff RS, Montori VM (2006). Testosterone therapy in adult men with androgen deficiency syndromes: an endocrine society clinical practice guideline. J Clin Endocrinol Metab.

[3] Anaissie J, DeLay KJ, Wang W, Hatzichristodoulou G, Hellstrom WJ (2017). Testosterone deficiency in adults and corresponding treatment patterns across the globe. Transl Androl Urol.

[4] Zucker IJ, Masterson TA (2021). Comparison of American Urological Association and Endocrine Society guidelines on testosterone replacement. Int J Impot Res.

[5] Madhusoodanan V, Patel P, Lima TF (2019). Human Chorionic Gonadotropin monotherapy for the treatment of hypogonadal symptoms in men with total testosterone >300 ng/dL. Int Braz J Urol.

[6] Best JC, Gonzalez D, Masterson TA, Blachman-Braun R, Pai R, Ramasamy R (2021). A cross-sectional comparison of secondary polycythemia in testosterone-deficient men treated with nasal testosterone gel vs. intramuscular testosterone cypionate. Can Urol Assoc J.

[7] Kim DK, Noh JW, Chang Y, Lee HY, Park JJ, Ryu S, Kim JH (2020). Association between prostate-specific antigen and serum testosterone: a systematic review and meta-analysis. Andrology.

[8] Vicari E, Mongioì A, Calogero AE, Moncada ML, Sidoti G, Polosa P, D'Agata R (1992). Therapy with human chorionic gonadotrophin alone induces spermatogenesis in men with isolated hypogonadotrophic hypogonadism - long-term follow-up. Int J Androl.

[9] Wenker EP, Dupree JM, Langille GM (2015). The use of hCG-based combination therapy for recovery of spermatogenesis after testosterone use. J Sex Med.

[10] Habous M, Giona S, Tealab A (2018). Clomiphene citrate and human chorionic gonadotropin are both effective in restoring testosterone in hypogonadism: a short-course randomized study. BJU Int.

[11] Lee JA, Ramasamy R (2018). Indications for the use of human chorionic gonadotropic hormone for the management of infertility in hypogonadal men. Transl Androl Urol.

[12] Soares AH, Horie NC, Chiang LA (2018). Effects of clomiphene citrate on male obesity-associated hypogonadism: a randomized, double-blind, placebo-controlled study. Int J Obes (Lond).

[13] Helo S, Ellen J, Mechlin C, Feustel P, Grossman M, Ditkoff E, McCullough A (2015). A randomized prospective double-blind comparison trial of clomiphene citrate and anastrozole in raising testosterone in hypogonadal infertile men. J Sex Med.

[14] Huijben M, Lock MT, de Kemp VF, de Kort LM, van Breda HM (2022). Clomiphene citrate for men with hypogonadism: a systematic review and meta-analysis. Andrology.

[15] Buvat J, Lemaire Lemaire, A A, Buvat-Herbaut M (1987). Human chorionic gonadotropin treatment of nonorganic erectile failure and lack of sexual desire: a double-blind study. Urology.

[16] Ishikawa T, Ooba T, Kondo Y, Yamaguchi K, Fujisawa M (2007). Assessment of gonadotropin therapy in male hypogonadotropic hypogonadism. Fertil Steril.

[17] Rainer Q, Pai R, Masterson T (2022). Safety of human chorionic gonadotropin monotherapy for men with previous exogenous testosterone use or replacement therapy. J Sex Med.

[18] Scovell JM, Ramasamy R, Wilken N, Kovac JR, Lipshultz LI (2015). Hypogonadal symptoms in young men are associated with a serum total testosterone threshold of 400 ng/dL. BJU Int.

